# Using Ecological and Twitter-Based Assessments to Examine Impacts in Temporal and Community Context

**DOI:** 10.1093/geroni/igab046.053

**Published:** 2021-12-17

**Authors:** Isabella Bouklas, Giselle Ferguson, Giancarlo Pasquini, Huy Vu, Mohammadzaman Zamani, Ruixue Zhaoyang, Stacey Scott, H Andrew Schwartz

**Affiliations:** 1 Duke University , Durham, New York, United States; 2 Stony Brook University, Stony Brook, New York, United States; 3 The Pennsylvania State University, University Park, Pennsylvania, United States

## Abstract

In March 2020, Bronx County (NY) saw one of the first U.S. COVID-19 outbreaks. This outbreak coincided with the ongoing Einstein Aging Study (EAS), which involved older adults living in Bronx County completing annual two-week intensive data collection “bursts.” Thus, it serves as a natural experiment to study pre-COVID to early pandemic-related changes in the daily well-being of participants who were at risk both due to their age and their location. We examined within-person change in self-reported negative thoughts, affect, stress, and loneliness from a subsample of 78 EAS participants. Participants’ data from a two-week “burst” of momentary surveys during 2019 were compared with their data from the corresponding timeframe during the early COVID-19 period (February-June 2020). Personality and mild cognitive impairment were examined as predictors of change. Average momentary loneliness significantly increased from 2019 to 2020. Participants with greater neuroticism increased more in thought unpleasantness and depressed feelings. To understand the community context, community distress markers were analyzed using Artificial Intelligence (AI)-based assessments of public Twitter posts from Bronx County during the same periods. These Twitter posts also showed a surge of COVID-related topics at the onset of the Bronx outbreak. Language analysis showed a 2019-2020 increase in Bronx community markers of anxiety, depressivity, and negatively-valenced affect extracted from Twitter. We observed 2019-2020 change in both individuals’ well-being (via intensive reports) and in their communities (via Twitter). Contextualizing these with the increased COVID-19 discussion online suggests that these may reflect common pandemic effects.

